# Synthesis of Icariin-Zinc and its Protective Effect on Exercise Fatigue and Reproductive System Related Glands in Male Rats

**DOI:** 10.3389/fphar.2021.611722

**Published:** 2021-06-09

**Authors:** Juntao Zhang, Chao Zhang, Aifeng Liu, Qiang Ji, Lixia Ren, Chuanrui Ma, Hengyu Zhang, Chaochao Wu, Donglin Zhang, Man Shang, Feng He

**Affiliations:** ^1^ Academy of Medical Engineering and Translational Medicine, Tianjin University, Tianjin, China; ^2^ Orthopedics Department, The First Teaching Hospital of Tianjin University of Traditional Chinese Medicine, Tianjin, China; ^3^ Graduate College, Tianjin University of Traditional Chinese Medicine, Tianjin, China; ^4^ School of Materials Science and Engineering, Tianjin University, Tianjin, China; ^5^ Department of Pharmacology, School of Basic Medical Sciences, Tianjin Medical University, Tianjin, China

**Keywords:** icariin-zinc, synthesis, protective effect, exercise fatigue, reproductive system

## Abstract

**Background:** Icariin, a traditional Chinese medicine, plays a protective role in the treatment of exercise fatigue. Zinc, a trace element, plays an important role in the reproductive system. Therefore, we aimed to synthesize an Icariin-Zinc complex (by chemical means) and verify its protective effect on exercise fatigue and the reproductive system using animal experiments.

**Methods:** The icariin-zinc complex was prepared by the reaction of icariin carbonyl and zinc ions (molar ratio 1:3). The molecular formula and structural formula of the complex were identified and tested. Fifty-six rats selected by swimming training were randomly divided into six groups: static control, exercise control, icariin, gluconate zinc (G-Zn group), icariin glucose zinc and icariin-zinc exercise ( low, high dose/L-E group, H-E group) groups. These groups respectively received the following doses: 1 ml/100 g, daily gavage with NS (for the first two groups), 45 mg/kg icariin, 110 mg/kg Gluconate Zinc, Icariin glucose zinc (45 mg/kg Icariin and 110 mg/kg Gluconate Zinc), 60 mg/kg icariin zinc and 180 mg/kg icariin zinc. After 3 weeks of gavage, we conducted 6 weeks of exhaustive swimming training. Test indices such as exhaustive swimming time of rats and body weight were evaluated after the last training exercise. The seminal vesicles, testes, and prostate gland were weighed, and their indices were calculated. The levels of testosterone (in the plasma) and glycogen (in the liver and muscle homogenates) were also evaluated using ELISA.

**Results:** Compared with the static control group, the exhaustive swimming time of the rats in each group was prolonged. Compared with the other groups, the exhaustive swimming time of the L-E and H-E groups was significantly longer (*p* < 0.01); the Icariin-Zinc complex significantly increased the exhaustive swimming time of the rats. Compared with the static control group, the plasma testosterone content of the L-E and H-E groups increased significantly (*p* < 0.05). Compared with the exercise control group and G-Zn group, the plasma testosterone content of the H-E group also increased significantly (*p* < 0.01). The Icariin-Zinc complex significantly increased the serum levels of testosterone in rats. Compared with the control group, the muscle glycogen reserves of each group decreased, indicating that the muscle glycogen reserves of the rats decreased after swimming. Compared with other groups, the Icariin-Zinc complex can reduce the level of glycogen in the muscles, indicating that it can increase the utilization efficiency of glycogen in muscles. Compared with the static control and exercise control groups, the testicular weight of rats in the administration groups increased slightly. The Icariin-Zinc complex increased the testicular weight, indicating that the function of the reproductive system was improved to some extent.

**Conclusion:** Icariin-Zinc can significantly prolong the exhaustive swimming time, improve exercise ability, and increase the plasma testosterone level (which is beneficial for improving the reproductive ability of male rats). Moreover, the beneficial effect of Icariin-Zinc on the glycogen content, testis index, and other reproductive system glands is dose-dependent.

## Background

Fatigue is a complex physiological and pathological that seriously affects human health. Epimedium is a perennial herb belonging to the family Berberidaceae. It has been used for thousands of years to promote male potency. Traditional Chinese medicines are believed to help in tonifying the kidneys and invigorating the Yang, and they have been used to treat impotence, premature ejaculation, lumbago and leg pain, neurasthenia, forgetfulness, and other diseases. Icariin (ICA) is the main active component extracted from Herba Epimedii (herbal medicine made from dried aerial parts of Epimedium). ICA has many functions such as neuroprotection, antitumoral activity, antioxidative activities, immunomodulation, and promotion of reproductive functions (especially in male mice). It has become a research hotspot at home and abroad (Liu et al., 2004; Tan et al., 2016).

Many in vivo studies have shown the beneficial effect of ICA on reproductive functions (Kang et al., 2012; Chen et al., 2014). ICA increases the epididymal sperm count and testosterone level in male rats (Chen et al., 2014). Mechanistically, the improved sexual function of male mice mediated by ICA might be associated with the hypothalamic-pituitary-gonadal axis and the PI3K/AKT/eNOS/NO signaling pathway (Ding et al., 2017). In addition, ICA could protect human sperm from being damaged by FeSO_4_/H_2_O_2_ (Zhan et al., 2014). It can maintain the “Raman fingerprint” of human sperm, suggesting that ICA may serve as a tonifying and replenishing agent of herbal origin that enhances reproductive functions. These show that ICA can promote the function of human and animal reproductive systems and provide a basis for animal experiments.

Zinc is a component of more than 300 types of metal enzymes in the human body (Hennigar and Kelleher, 2012). Zinc is reported to be an essential element for the normal function of the male reproductive system and sperm. It plays an important role in the production, storage, secretion, and function of male sex hormones (Chu et al., 2016). Studies have shown that there is a relationship between sexual dysfunction and physical fatigue (Young et al., 2017; Fairbanks et al., 2017).

Based on the protective effect of Icariin and Zinc on the reproductive system, we hypothesized that an Icariin-Zinc combination might have a synergistic protective effect on the reproductive system and exercise fatigue.

## Methods

### Experimental Animals and Drugs

All procedures were performed in accordance with the NIH guidelines for the Care and Use of Laboratory Animals (NIH Publications No. 80–23, revised 1996), and the research protocol was approved by the Ethics Committee of Tianjin University of Traditional Chinese Medicine (TCM-LAEC2019069). Sixty-six Wistar male rats weighing 200–220 g were obtained from the Academy of Military Medical Science in Beijing and housed in special cages in a specific-pathogen-free (SPF) environment, allowing free movement and ad libitum access to food and drink. Icariin (purity ≥ 98%, Lot No. 181209, Shanghai Mochi Biotechnology Co., Ltd.), zinc chloride (GR, lot No. G1820107, Shanghai Alading Biochemical Technology Co., Ltd.), zinc reagent (AR, H08F9S54539, Shanghai Yuanye Biotechnology Co., Ltd.), distilled water, anhydrous ethanol (Shandong lierkang Medical Technology Co., Ltd.), 75% ethanol (Shandong lierkang Medical Technology Co., Ltd.), and 0.9% sodium chloride (NS, batch number 19012523, China Otsuka Pharmaceutical Co., Ltd.) were the main chemicals used in the experiment.

### Experimental Instruments

The main instruments used in the experiment were a constant temperature magnetic stirrer (78HW-1, Hangzhou instrument and motor company), a rotary vaporizer (re-2000e, Gongyi Yuhua Instrument Co., Ltd.), a circulating water multi-purpose vacuum pump (SHB-3, Gongyi Yuhua Instrument Co., Ltd.), an electronic analysis balance (BS210s, Beijing Saiduolis Balance Co., Ltd.), mobile phone timer (COR-Al10, honor), swimming sink (L×W×H=100 × 50 × 60 cm), and a water pump (Jiabao “AP1500” type).

### Preparation and Detection of the Icariin-Zinc Complex

#### Preparation and Extraction of Icariin-Zinc

Three thousand milligrams of icariin and 1200 mg of zinc chloride were mixed (presumably the molar ratio of icariin to zinc chloride is 2:1 or 1:1 in theory). To make icariin fully react, 1:2.5 mixture ratio was selected and dissolved in 500 ml (volume fraction is 45%) of ethanol solution, and a constant temperature magnetic stirrer was used (the parameters were set at 400 rpm, 70°C [range 60–80°C] oil bath) to stir the mixture for at least 24 h. The reacting solution was then placed in a water bath at 45°C. The final icariin-zinc product was obtained by centrifugation and was repeatedly washed with pure water to remove unreacted icariin.

### Preparation of the Zinc Reagent Solution

Zinc reagent (0.1 mg) was weighed and dissolved in 125 ml anhydrous ethanol. After it had completely dissolved, it was stored in a brown bottle.

### Identification of Compounds (Colorimetry)

The reaction between the zinc reagent solution and zinc ion was used for identification. The reactant (that is, final icariin-zinc product) was completely dissolved in anhydrous ethanol after the last washing. Adding the zinc reagent solution to it turned the color of the mixture blue; meanwhile, adding the zinc reagent solution to the last washing solution did not change its color to blue. As such, it could be concluded that the reactant was the icariin-zinc complex and that there was no zinc chloride residue in the reactant.

### Molecular Formula and Structural Formula of the Icariin-Zinc Complex

#### Sample Pretreatment

A 0.05-g sample of the compound was placed in a microwave digestion tank made up of polytetrafluorethylene. The sample was soaked for 2 h in mixture of 6 ml of nitric acid and 1 ml of hydrogen peroxide. Subsequently, it was digested for 20 min at 190°C. Acid removal, constant volume to 100 ml. This needs to be determined. ICP-OES on-line measurement: A VISTA-MPX inductively coupled plasma spectrometer (Varian Company, United States), referred to as ICP-OES was used to determine the formula (see Table 1).

**TABLE 1:** ICP-OES Instrument Operating Conditions.

Working Parameters	Setting Conditions	Working Parameters	Setting Conditions
Power	1200 W	Reading stability delay	10 s
Cooling gas (AR) flow	15.0 L/min	Injection stability delay	20 s
Auxiliary gas (AR) flow	1.5 L/min	Pump speed	15 rpm
Atomizing gas (AR) pressure	200 kPa	Cleaning time	310 s
Observation height	10 mm	Number of readings	3times
Reading time	10 s		

The measured wavelength was 213 nm. The average of three results were taken.

**FIGURE 1:**
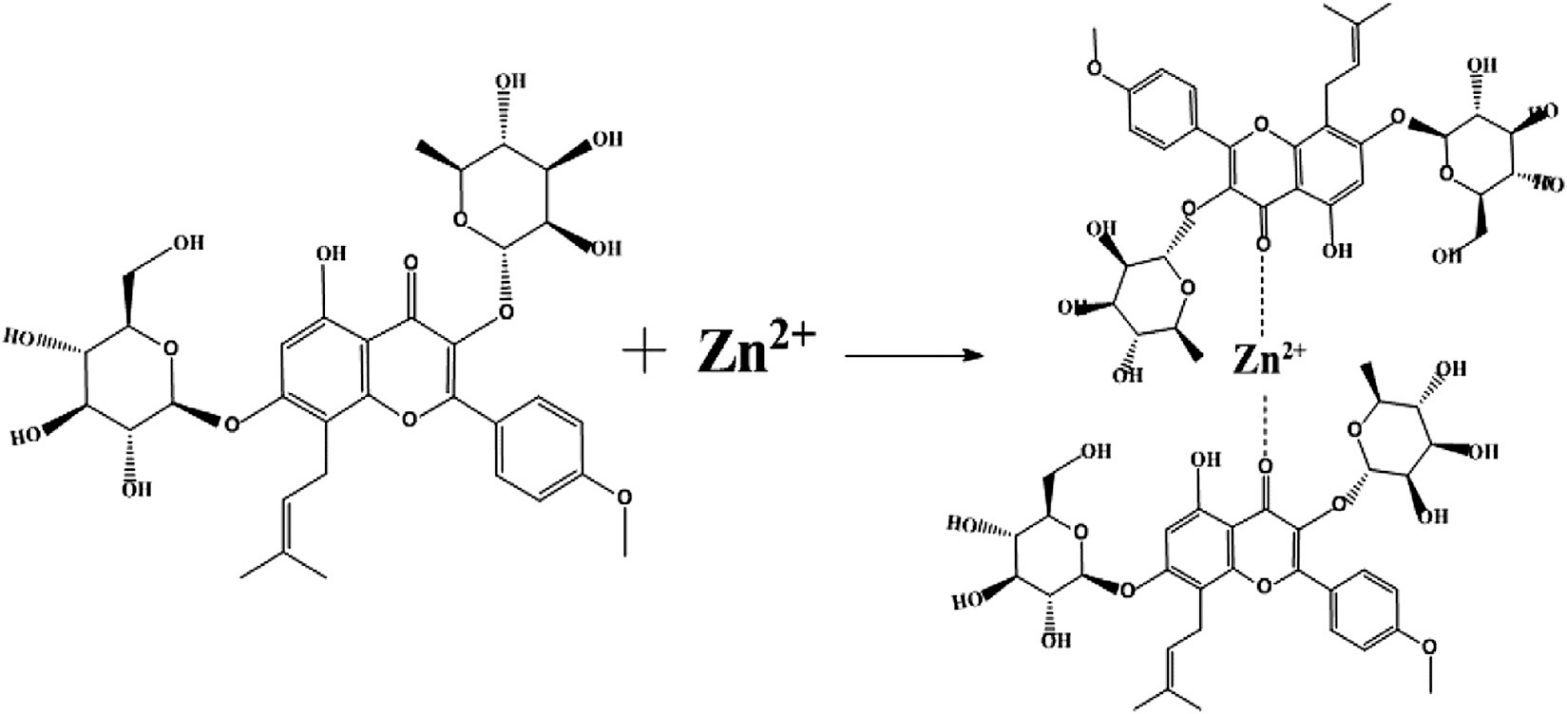
Chemical Synthesis Route of the Icariin-Zinc complex.

The Zn^2+^ content was 4.6%. Combining the structural formula and molecular weight of icariin, we found that icariin and Zn are 2-coordinated (that is, two carbonyls (C=O) and one Zn^2+^ coordinate). The molecular formula is (C_33_H_40_0_15_)_2_Zn^2+^, with a relative molecular weight of 1417 (Figure 1).

### Experimental Groups

After 3 days of adaptive feeding and 3 days of swimming training (the rats were trained 20 min/d), the rats were randomly divided into 7 groups with 8 rats in each group. Each group received a basic feeding and distilled water, and was fed freely every day. Each group was treated with different doses of 1 ml/100 g by gavage for 3 weeks (see Table 2, Experimental grouping and intervention).

**TABLE 2:** Experimental grouping and intervention.

Grouping	Intervention measures
Control group	Normal saline
Exercise group	Normal saline
Icariin group	45 mg/kg Icariin
G-Zn group	110 mg/kg Gluconate Zinc
Icariin glucose zinc	45 mg/kg Icariin, 110 mg/kg Gluconate Zinc
Low dose Icariin zinc exercise group (L-E group)	60 mg/kg Icariin zinc
High dose Icariin zinc exercise group (H-E group)	180 mg/kg Icariin zinc

### Anti-Fatigue Experimental Method (Surhio et al., 2017)

The control group did not undergo any training. In the other groups, a swimming trough of 100 × 50 × 60 cm was used as the swimming training device for the rats, and the water depth was set at 50 cm. The water temperature was 31 ± 2°C, and a water pump was installed at the bottom of the water tank to cause the water to flow (the aim was to prevent rats from floating on the water surface). Swimming training to exhaustion was performed every week for a total of 6 weeks. The parameters were changed every week. The first week, the rats exercised without weight, the second week with 2% weight, the third week with 4% weight, and the fourth to the sixth week with 5% weight. Each time, the swimming training was performed to exhaustion. The last time, all groups performed exhaustion swimming training without weight, and the time of swimming before exhaustion was measured. The time from the beginning of swimming to exhaustion which is a measure of the exhaustion exercise ability, was used as an index of anti-exercise fatigue ability. The standard used to determine exhaustion was the inability of the rats to show up on the water surface 10 seconds after sinking.

### Test Indices and Methods of Measurement (Xi et al., 2018)

Each group was weighed 24 h after the last training. Blood samples were taken from the inner canthi of the rats and sodium citrate solution added to prevent coagulation. The samples were incubated in a 37°C water bath for 30 min, centrifuged at 3000 R/min for 10 min, and plasma was separated. The plasma testosterone levels were measured.

All rats were administered with an overdose of 2,2,2-tribromoethanol (640 mg/kg, intraperitoneal injection) for anesthesia. The seminal vesicles, testes, prostate, liver, and the deep quadriceps femoris muscles (without the fascia) were removed. These organs were washed in precooled normal saline to remove the blood stains, dried with filter paper, and stored in a refrigerator at −20°C. The seminal vesicle, testis, and prostate glands were weighed, and their indices (the ratio of seminal vesicle gland, testis, and prostate gland weights to body weight) were calculated.

To prepare the liver and muscle tissue homogenate, appropriate amounts of tissues (0.2−1 g) and precooled normal saline (NS) were added into a beaker in a tissue mass (g) to liquid volume (ml) homogenate medium proportion of 1/9. The tissue was then quickly cut into pieces (the above operations were carried out in an iced water bath). The supernatant was separated and extracted by centrifugation at 4°C, 3000 rpm for 15 min and then refrigerated at 4°C or frozen at −20°C for standard. The supernatant was used to measure the glycogen content of the liver or muscle homogenate.The remaining mice were killed (anesthetized and euthanized in a CO_2_ chamber).

## Results

### Observation of the Feeding and Swimming Behavior

The rats in each group were fed for 6 weeks. At the third week, the rats in each group were more excited, the amount of activity in the cages increased, the fighting phenomenon appeared, the number of squeaks increased, their voices became louder, and they struggled more strongly when grasped and during weighing. The amount of food and drinking water given to the icariin-zinc group and the exercise control group was slightly more than that in the static control group. The swimming abilities of the rats in each group were observed, and the rats in the icariin-zinc group were found to be more active, to jump out of the water and squeak more frequently, and their relative swimming distances were longer.

### Effect of Icariin-Zinc on Body Weight of Rats

**FIGURE 2:**
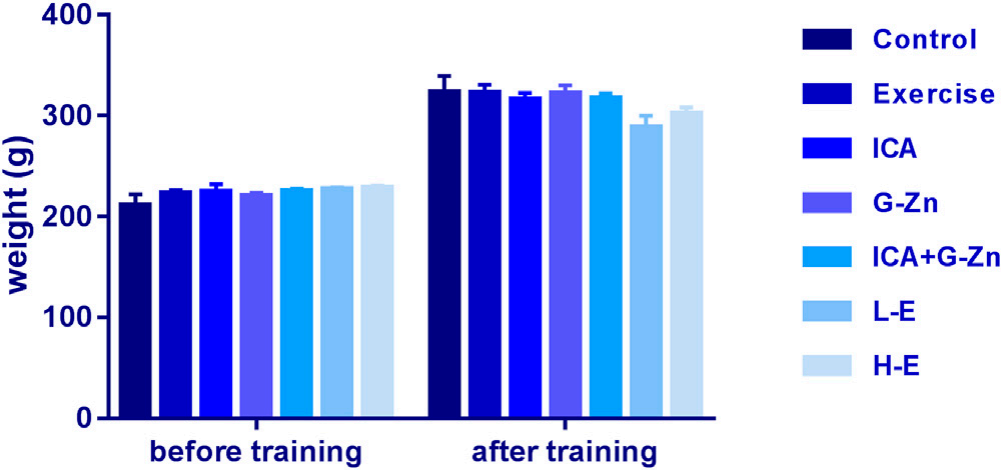
Effect of icariin-zinc on body weight of rats (*n* = 8, mean ± SD).

Before the beginning of the experiment, the average body weight of the rats in each group was approximately 250 g. After continuous feeding for 3 weeks, the average body weight of rats increased steadily to approximately 320 g, and there was no significant difference in body weight between the three groups. After 6 weeks of weight-bearing swimming training, the body weight was taken before the last exhaustive swimming. The average body weight of the static control group had increased by about 4% meanwhile that of the exercise control group had reduced. After the administration of the different concentrations of icariin-zinc, the average body weight of rats in the L-E and H-E groups decreased compared with the exercise control group (Figure 2).

### The Effect of Icariin-Zinc on Anti-Fatigue in Rats

**FIGURE 3:**
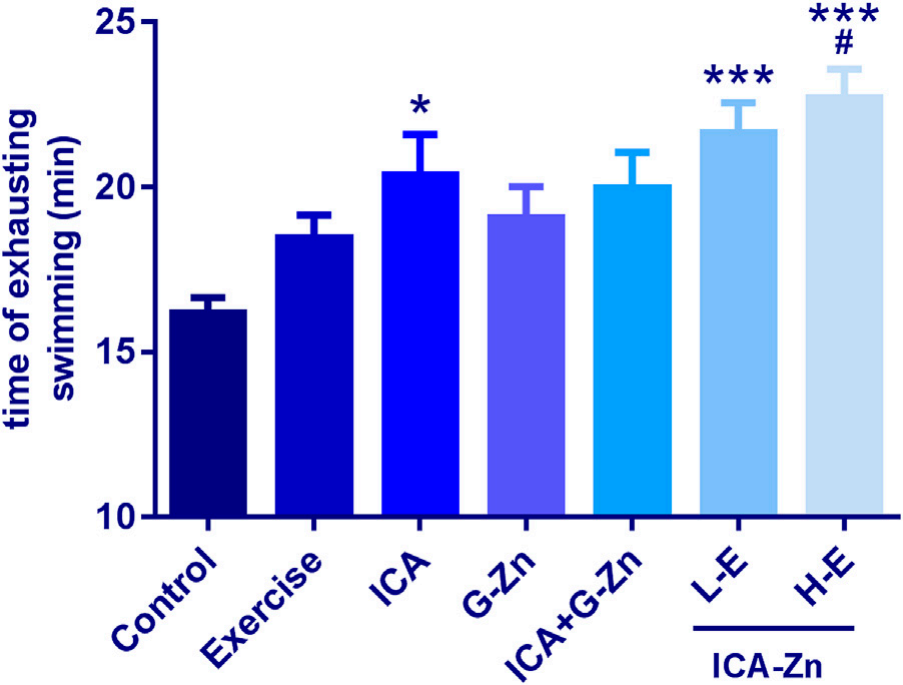
Effect of icariin-zinc on anti-fatigue of rats (*n* = 8, mean ± SD). *p ＜ 0.01, ***p ＜ 0.001 vs. Control；^*#*^ *p* ＜ 0.05 vs. Exercise.

Compared with the static control, the exhaustive swimming time of rats in each group was prolonged. After administration of the icariin-zinc, the exhaustive swimming times of the L-E and H-E groups were significantly prolonged (*p* <0.01), just as that of the ICA group (Figure 3). It is suggested that icariin-zinc has the same function as ICA in improving the exercise ability of rats. The swimming abilities of rats in each group were observed, and the rats in the icariin-zinc group were more active, jumped out of the water more frequently during swimming, had a longer relative swimming distance, and had a more prolonged exhaustive swimming time. These showed that the physical functional reserves of the rats were better and their exercise ability was enhanced.

### The Effect of Exercise and Icariin-Zinc on Glycogen Storage in the Liver and Muscle of Rats

**FIGURE 4:**
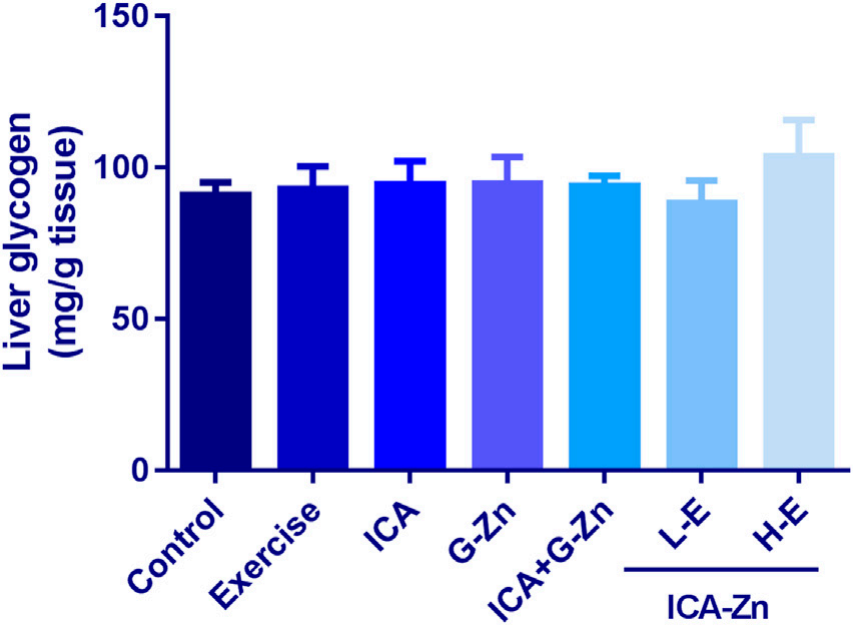
Effect of icariin-zinc on liver glycogen level in rats (*n* = 8, mean ± SD).

**FIGURE 5:**
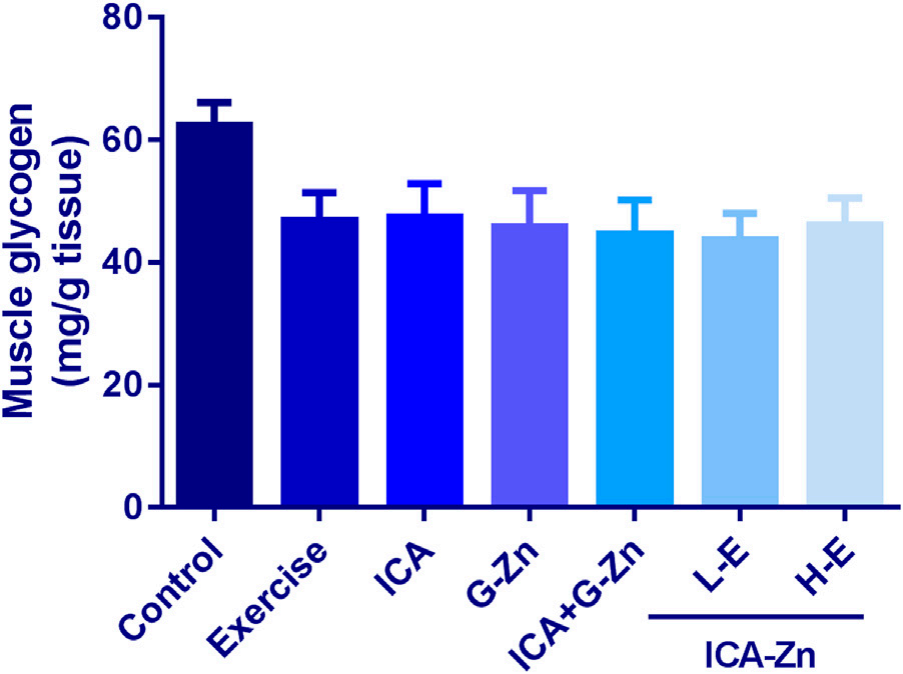
Effect of icariin-zinc on muscle glycogen level in rats (*n* = 8, mean ± SD).

The content of liver glycogen was the same in all the groups but for the H-E group there was an increasing trend (Figure 4). Compared with the control group, the muscle glycogen reserves of each group decreased after swimming (Figure 5). A comparative evaluation showed that icariin-zinc had no significant effect on liver glycogen and muscle glycogen storage in rats.

**FIGURE 6:**
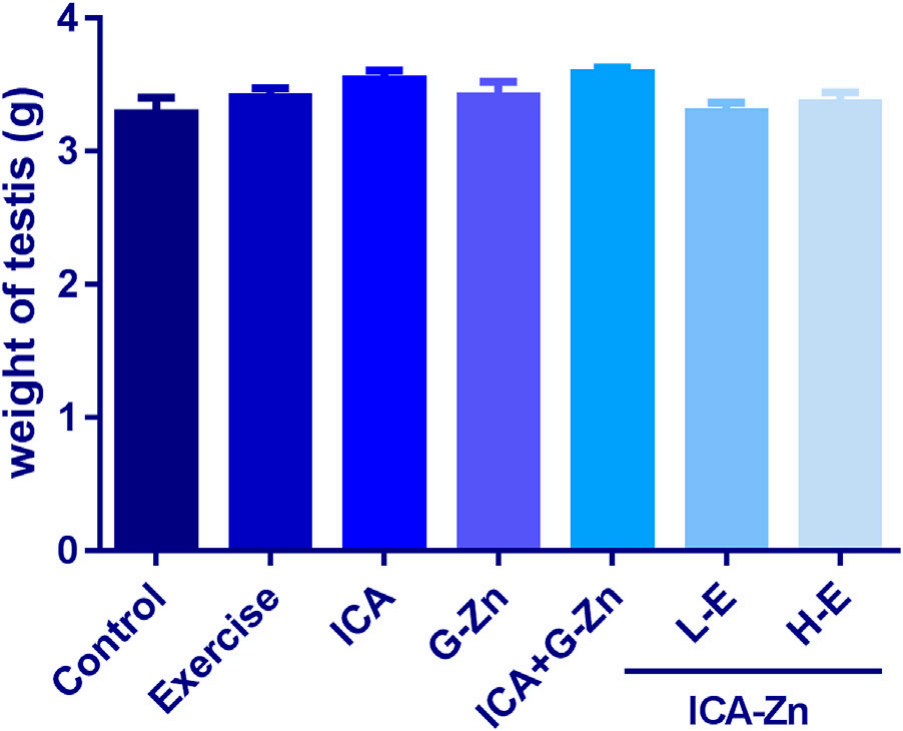
Effect of icariin-zinc on testis weight in rats (*n* = 8, mean ± SD).

**FIGURE 7:**
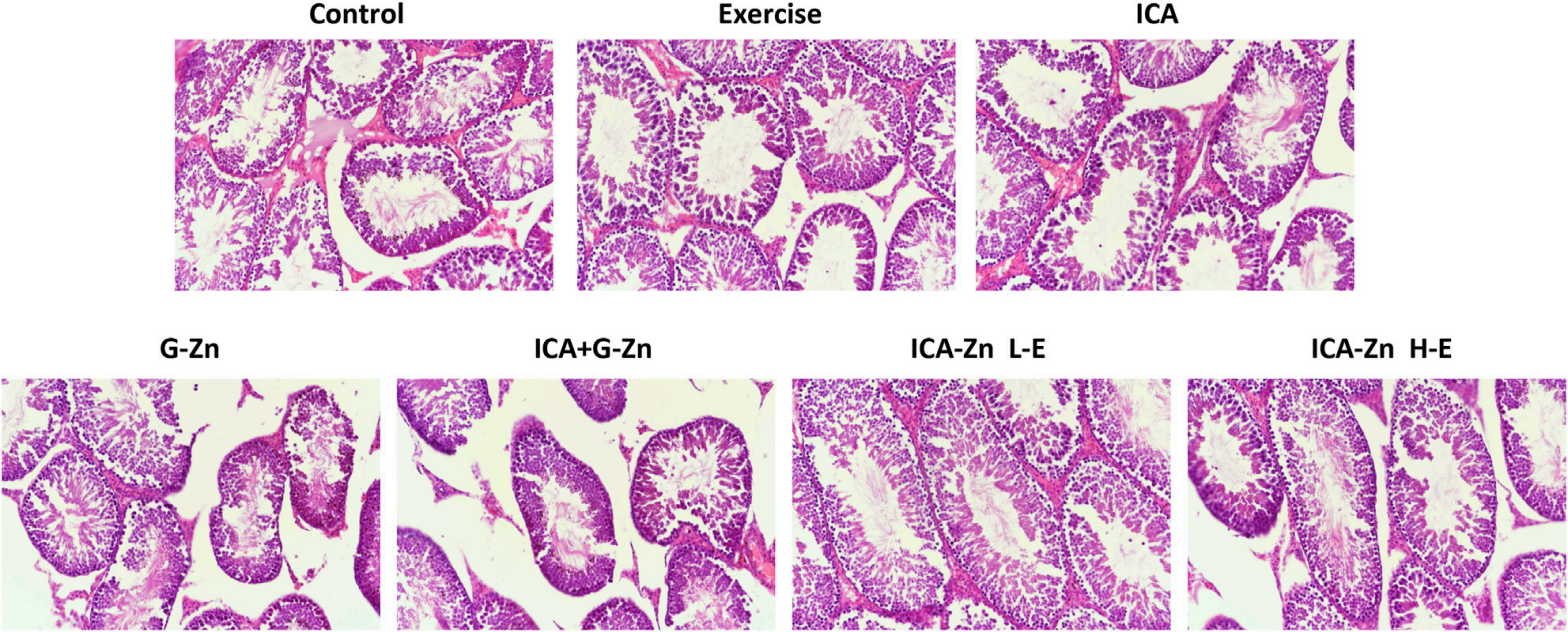
HE staining of testes in rats (200x).

There was no significant difference between the testicular weights of the different groups when compared with the control (Figure 6). Hematoxylin and eosin (HE) staining showed normal morphologies of Leydig and Sertoli cells, continuous and complete membrane of the seminiferous tubules, and a regular arrangement of germ cells. These findings suggest that icariin-zinc does not affect testes histopathology (Figure 7).

### The Effect of Icariin-Zinc on the Testicular Weight and Index of Rats 

**FIGURE 8:**
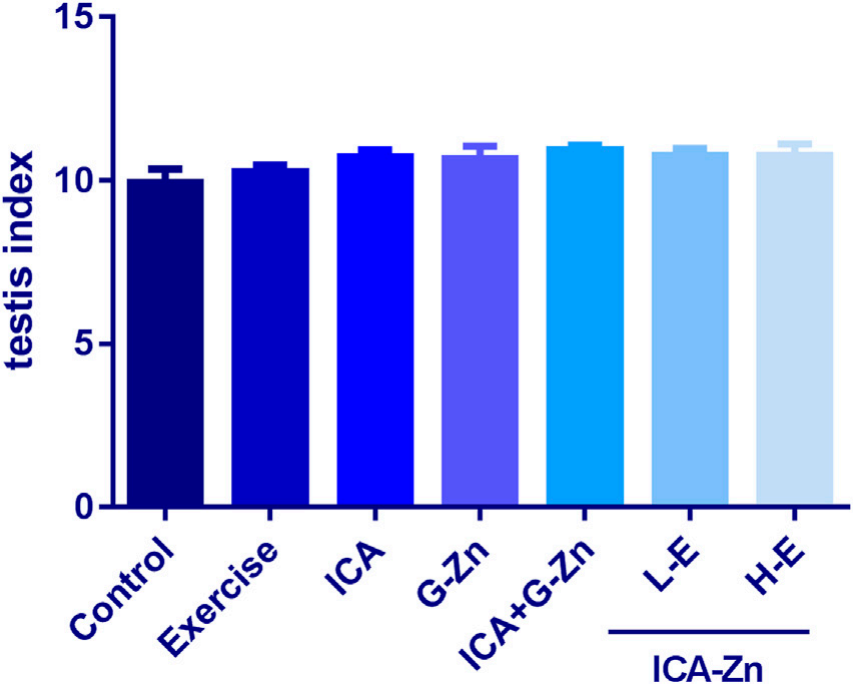
Effect of icariin-zinc on testicular index of rats (*n* = 8, mean ± SD).

Compared with the static control group and exercise control group, the testicular index of rats in the treatment group increased slightly (Figure 8). Testicular index = testis/weight x 1000. Testicular index measurement helps to eliminate the influence of animal weight, and can be used to evaluate the function of the rat’s reproductive system. The results showed that after intragastric administration of ICA, G-Zn, icariin glucose, zinc, and icariin-zinc, the testicular index increased, indicating that icariin-zinc could improve reproductive function in male rats.

### The Effect of Exercise and Icariin-Zinc on the Weights of the Seminal Vesicle and Prostate Gland in Rats

**FIGURE 9:**
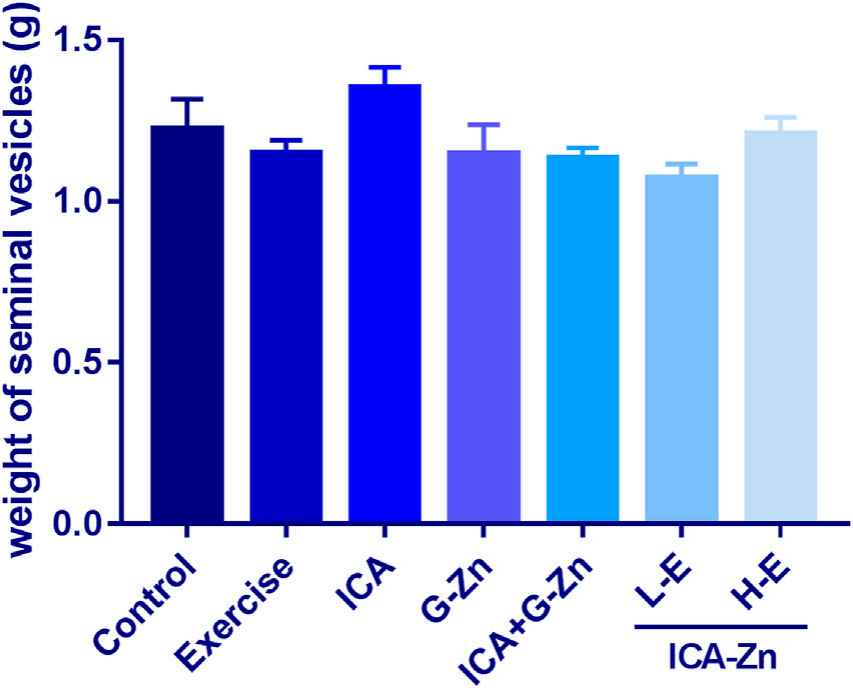
Effect of icariin-zinc on the weight of the seminal vesicle weight in rats (*n* = 8, mean ± SD).

**FIGURE 10:**
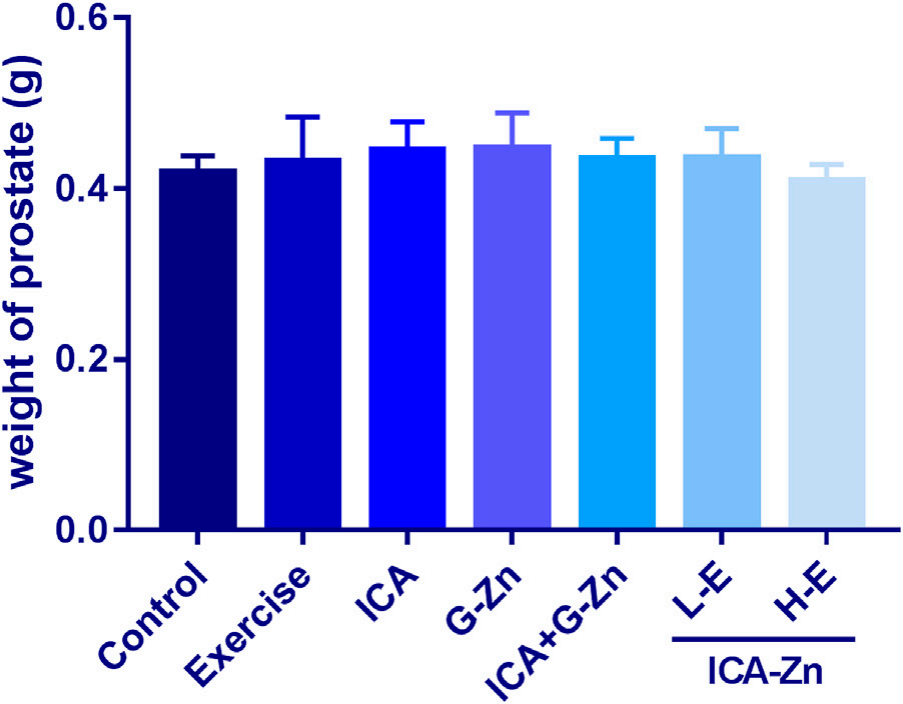
Effect of icariin zinc on the weight of the prostate gland in rats (*n* = 8, mean ± SD).

Compared with the exercise control group, the average weight of the seminal vesicles of rats in the L-E group increased significantly, indicating that the sperm reserve of the rats in the L-E group was larger. This phenomenon was not observed in the HE group (Figure 9). However, icariin-zinc had no significant effect on prostate weight in rats (Figure 10).

### The Effect of Exercise and Icariin-Zinc on the Plasma Testosterone Level in Rats 

**FIGURE 11:**
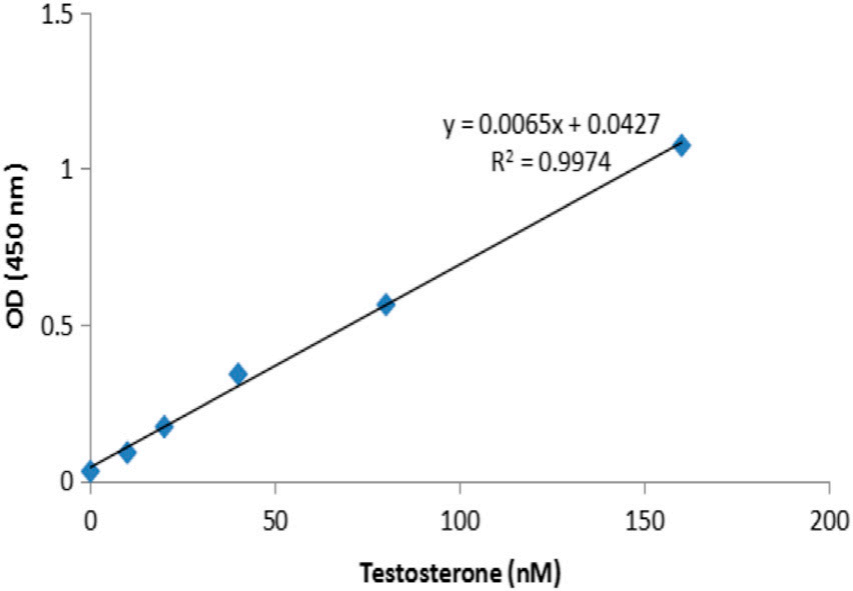
Testosterone standard curve.

**FIGURE 12:**
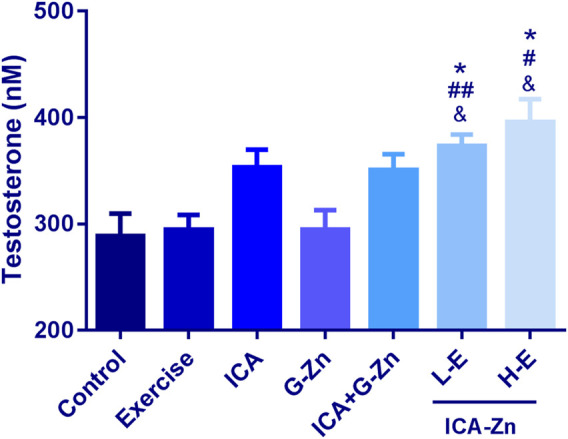
Effect of exercise and icariin-zinc on the plasma testosterone content of rats (*n* = 8, mean ± SD). *p < 0.05 vs. Control; ^*#*^ *p <* 0.05, ^*##*^ *p <* 0.01 vs. exercise; ^*&*^ *p <* 0.05, ^*&&*^ *p <* 0.01 vs. G-Zn.

The concentration of testosterone in the plasma of rats was measured by ELISA, and the standard curve was obtained with good correlation (r = 0.9985) (Figure 11). Compared with the static control group, the plasma testosterone content of the L-E and H-E groups increased significantly (*p*< 0.05). Compared with the exercise control group and G-Zn group, the plasma testosterone content of the H-E group also increased significantly (*p*< 0.01). The above experimental results show that icariin-zinc can significantly improve the plasma testosterone level in rats, largely than icariin alone and zinc agents such as zinc gluconate (Figure 12).

## Discussion

**FIGURE 13:**
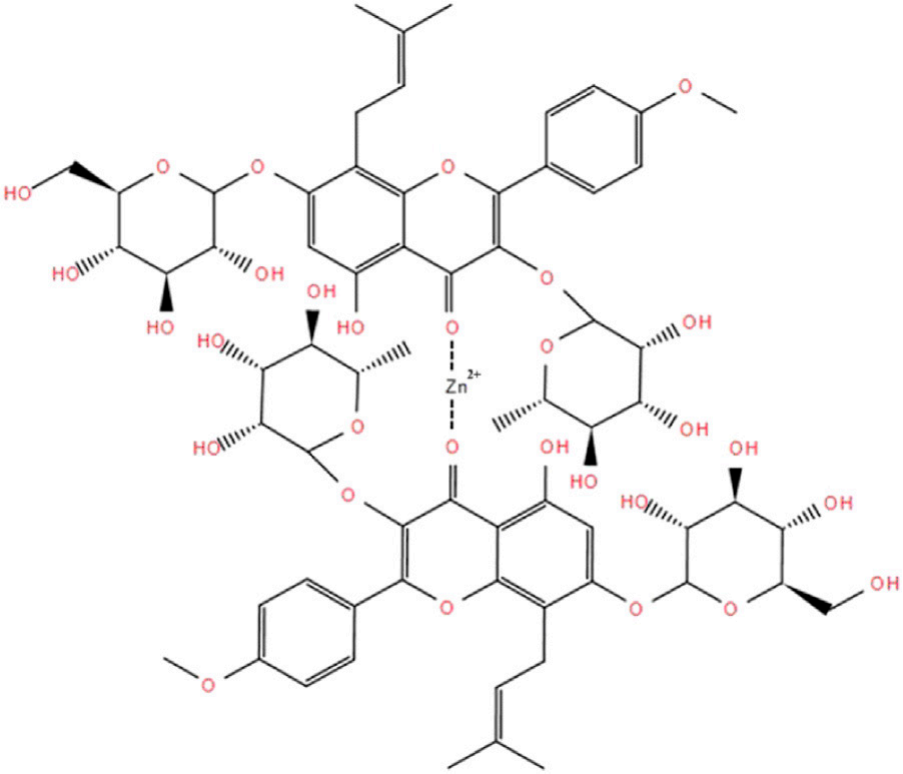
Molecular structure of Icariin-Zinc.

Icariin is the main extract and active component of the herbaceous plant epimedium which among others, plays a role in tonifying the kidney, strengthening the yang, and slowing down aging. Longh et al. (2018) found that icariin improved erectile function of SHR (Long et al., 2018). Zinc is a part of the structural composition of many proteins. It helps in the recovery of the nervous system. Zinc also plays an important biological role in male reproductive physiology and endocrine system (Santos and Teixeira, 2019). In this study, icariin and zinc were combined to produce a synergistic effect. Icariin-zinc was prepared by chemical synthesis. It was formed by the combination of two molecules of icariin with one molecule of divalent zinc ion through ionic and coordination bonds (Figure 13). The hydroxyl group provides an electron pair and forms a coordination bond with zinc, giving the compound a good chemical stability. After chemical synthesis, the structure of each particle is the same, uniform, and easy to absorb. Physical mixing has different rules, and makes it difficult to achieve a uniform mixing proportion thus impacting the experimental data. Through the study of its effects on body weight, exercise exhaustive time, testicular and seminal vesicle weight, and glycogen storage, we were able to put into evidence preliminary improvements in anti-fatigue and sexual function in rats.

Body weight is an important indicator of the skeletal, muscular, and functional states, and overall development of the body. A change in body weight can reflect the adaptation of the body to sports training and the degree of its influence on the body. It is one of the most important indicators of animal growth and nutritional state. Our results showed that there was no significant difference between the body weights of the rats in the different groups during the first three weeks. It was considered that the normal development of the body weight had occurred. At the end of the 6th week, the body weight of rats in the L-E and H-E groups decreased, which was due to the increase in excitability and activity. In the process of feeding, the rats in the icariin-zinc group began to show hyperactivities (increased activity, fighting, and frequent squeaking) compared with the exercise control group and the static control group during the third week. This indicates that icariin-zinc can improve the excitability and exercise ability of male rats. The rats' body weight change, and daily amounts of food and water consumed were similar in both the icariin-zinc and exercise control groups. The change in body weight was slightly higher in the control group. This was because the compound promotes the development of the body, increases the basal metabolic rate, appetite, and amount of exercise.

Exercise fatigue is the physiological decline in working ability, activity ability, and range of activity after a strenuous exercise (Narkhede et al., 2016; Górski et al., 2017). The recovery time of exercise fatigue is relatively long and this has an impact on human life and work. Exercise exhaustive time is an important sign reflecting the body’s exercise ability. It can be used as an index to measure the anti-exercise fatigue ability of animals.

The exhaustion training resulted in the maintenance of the rats in both the icariin-zinc and exercise control groups in a state of exercise fatigue, and so, the exercise function could not be effectively recovered. Theoretically, in a short period of time, the exhaustion time would be shorter than that of the control group. The results of the anti-fatigue experiment showed that the exhaustive swimming time of rats in the exercise control group was shortened as expected. However, after the administration of icariin-zinc, the exhaustive swimming time of the L-E group was prolonged. Considering the hyperexcited state of the H-E group, the rats in the exercise control group struggled more violently during the exhaustion swimming training, jumped out of the water more frequently incurring great energy losses, and failed to rest for a long period of time. Because they could not store enough energy for exhaustive training, the both H-E and L-E groups exhaustive times were not prolonged. Our results showed that the concentration of the icariin-zinc was very important, and high concentrations would cause high excitability, which was not conducive for physical recovery. Low concentration can enhance the anti-exercise fatigue abilities, a consequence of the improvement of the exercise level in skeletal muscles.

Glycogen is a form of energy storage in organisms. Glycogen in the skeletal muscle is an important form of energy storage. In long-term aerobic exercise, muscle glycogen consumption often occurs and is related to the inability to maintain muscle contractility (Britto et al., 2018). In long-term and high-intensity sports, the storage of muscle glycogen before exercise determines the time of exhaustion, which directly affects endurance training and competition. The importance of glycogen in exercise ability is reflected in endurance exercise. During exercise, glycogen decomposition is accelerated, and glucose is released into blood by the liver to maintain blood glucose balance. Our results showed that after swimming, the glycogen reserves in the liver and muscle of rats in the exercise control group decreased to the lowest level. However, the glycogen reserves increased in each group after the administration of different concentrations of icariin-zinc, especially in the H-E group.

These results indicate that icariin-zinc can promote glycogen synthesis in rats undergoing training. The possible mechanism is that icariin-zinc can enhance the oxidation and absorption of glucose, thus promoting the synthesis of muscle glycogen and liver glycogen.

The testis is a male internal reproductive organ and plays a role in the development of male secondary sexual characteristics and physiological function. The seminal vesicle is an important accessory gland of men, and is involved in the process of semen accumulation and release. Seminal vesicle secretion accounts for about 50–80% of the volume of semen, and thus closely influences the quality and quantity of sperm (Zhao et al., 2019). Our results showed that the testicular weights and indices of the L-E and H-E groups were increased. This could be because icariin-zinc promotes testicular development and spermatogenesis. We found that, compared with other administration groups, the weight of the seminal vesicle of rats in the L-E group increased significantly. This was probably due to the increase in seminal vesicle secretion caused by Icariin-Zinc.

Testosterone is one of the main components of androgen. It promotes spermatogenesis, stimulates the growth and development of reproductive organs, maintains normal sexual desire, and promote protein synthesis. From this experiment, we found that icariin-zinc can significantly improve the plasma testosterone level of rats, and this effect is significantly better than when icariin and zinc agents, such as zinc gluconate, are administered separately. It is suggested that ICA can promote the secretion of testosterone. ICA is a flavonoid that can protect the synthesis of testosterone in mouse Leydig cells (by the effects of 2-ethylhexyl phthalate). It protects mouse testes from the damage induced by DEHP by blocking ROS and promoting the secretion of testosterone (Sun et al., 2019). It can be concluded that icariin-zinc may promote testosterone secretion by protecting rat testicular cells from damage.

To sum up, sports fatigue and men’s health problems are of great concern, and they often affect each other. Male sexual dysfunction can cause other functional abnormalities in the body and thus, influence mental and physical health. Hence, it is a problem that requires an urgent solution. Therefore, it will be of great significance if a fast and effective drug that improves anti-fatigue and sexual abilities was found. Based on the behavior of animals in this study, and the analyses of specific experimental data, we believe that the combination of icariin and zinc ion can improve the motor and anti-fatigue abilities of male rats (through the improvement of the functions of skeletal muscles and joint). In addition, it can promote the index and function of the glands of the reproductive system (especially increase in testicular and seminal vesicle weights, increase the glycogen reserve in rats, and improve exercise ability). The mechanisms of action may be related to zinc induced oxidative stress mechanism, or improvement of sexual function by the regulation of the hypothalamic pituitary gonadal axis and the PI3K/Akt/eNOS/ NO signal pathway by ICA (Ding et al., 2018). In the future, these mechanisms will be further studied in terms of enhancing testicular function, including steroid production, sperm count, sperm motility, and spermatogenesis in male rats. Therefore, the preparation and application of icariin-zinc provides a new idea to explore for increasing anti-fatigue ability, enhancing exercise ability, and solving male reproductive health problems.

## Conclusion

In this experiment, icariin-zinc was successfully prepared, and the reaction rate was positively related to the reaction temperature, reactant concentration, and reaction time. An appropriate amount of icariin-zinc compounds can promote development in male rats, improve their excitability, promote their recovery from the fatigue state, and enhance their anti-fatigue ability.

Icariin-zinc can significantly prolong the exhaustive swimming time, improve the exercise ability, and increase the content of testosterone in the plasma of male rats. However, there was no significant effect on glycogen content, testicular index, and other reproductive glands (such as the prostate gland and seminal vesicle). Moreover, icariin-zinc was better than zinc gluconate in prolonging exhaustive swimming time, and increasing plasma testosterone content. Compared with icariin, the effect of high-dose zinc is more significant. It is suggested that in the application of the icariin-zinc complex, the dosage should be strictly controlled to obtain the best drug effect without affecting the reproductive system organs.

## Data Availability

The original contributions presented in the study are included in the article/Supplementary Material, further inquiries can be directed to the corresponding authors.

## Abbreviations

ICA: icariin;; ICP-OES: inductively coupled plasma-optic emission spectrometer; NS: normal saline;; ELISA: enzyme linked immunosorbent assay; H&E: hematoxylin & eosin;; SD: standard deviation;; SPF: specific pathogen free;; ROS: reactive oxygen species.
